# Cardiovascular and autonomic dysfunction in long-COVID syndrome and the potential role of non-invasive therapeutic strategies on cardiovascular outcomes

**DOI:** 10.3389/fmed.2022.1095249

**Published:** 2023-01-19

**Authors:** Francisca J. Allendes, Hugo S. Díaz, Fernando C. Ortiz, Noah J. Marcus, Rodrigo Quintanilla, Nibaldo C. Inestrosa, Rodrigo Del Rio

**Affiliations:** ^1^Laboratory Cardiorespiratory Control, Department of Physiology, Pontificia Universidad Católica de Chile, Santiago, Chile; ^2^Instituto de Ciencias Biomédicas, Facultad de Ciencias de Salud, Universidad Autónoma de Chile, Santiago, Chile; ^3^Departamento de Biología, Mechanisms of Myelin Formation and Repair Laboratory, Facultad de Química y Biología, Universidad de Santiago de Chile, Santiago, Chile; ^4^Department of Physiology and Pharmacology, Des Moines University, Des Moines, IA, United States; ^5^Centro de Excelencia en Biomedicina de Magallanes, Universidad de Magallanes, Punta Arenas, Chile

**Keywords:** COVID-19, long-COVID, cardiovascular dysfunction, autonomic impairment, therapeutic strategy, cardiovascular outcomes, autonomic dysfunction

## Abstract

A significant percentage of COVID-19 survivors develop long-lasting cardiovascular sequelae linked to autonomic nervous system dysfunction, including fatigue, arrhythmias, and hypertension. This post-COVID-19 cardiovascular syndrome is one facet of “long-COVID,” generally defined as long-term health problems persisting/appearing after the typical recovery period of COVID-19. Despite the fact that this syndrome is not fully understood, it is urgent to develop strategies for diagnosing/managing long-COVID due to the immense potential for future disease burden. New diagnostic/therapeutic tools should provide health personnel with the ability to manage the consequences of long-COVID and preserve/improve patient quality of life. It has been shown that cardiovascular rehabilitation programs (CRPs) stimulate the parasympathetic nervous system, improve cardiorespiratory fitness (CRF), and reduce cardiovascular risk factors, hospitalization rates, and cognitive impairment in patients suffering from cardiovascular diseases. Given their efficacy in improving patient outcomes, CRPs may have salutary potential for the treatment of cardiovascular sequelae of long-COVID. Indeed, there are several public and private initiatives testing the potential of CRPs in treating fatigue and dysautonomia in long-COVID subjects. The application of these established rehabilitation techniques to COVID-19 cardiovascular syndrome represents a promising approach to improving functional capacity and quality of life. In this brief review, we will focus on the long-lasting cardiovascular and autonomic sequelae occurring after COVID-19 infection, as well as exploring the potential of classic and novel CRPs for managing COVID-19 cardiovascular syndrome. Finally, we expect this review will encourage health care professionals and private/public health organizations to evaluate/implement non-invasive techniques for the management of COVID-19 cardiovascular sequalae.

## 1. Introduction

The acute phase of the COVID-19 pandemic has tested health systems around the world. While respiratory aspects of COVID-19 have rightfully taken a primary focus in patient management due to their critical nature, it is worth emphasizing that COVID-19 also has potentially profound effects on cardiovascular, hepatic, renal, gastrointestinal, neurological, and metabolic function. Recent studies and meta-analyses show that there are sequelae of this disease that persist beyond the typical post-viral recovery period (1–3). According to the WHO, this “long form” of COVID-19 disease is defined as a “*condition that occurs in individuals with a history of probable or confirmed SARS-CoV-2 infection, usually 3 months from the onset of COVID-19 with symptoms that last for at least 2 months and cannot be explained by an alternative diagnosis. Common symptoms include fatigue, shortness of breath, and cognitive dysfunction, but may include others and are generally associated with an adverse impact on everyday function. Symptoms may be new onset following initial recovery from an acute COVID-19 episode or persist from the initial illness. Symptoms may also fluctuate or relapse over time*” (4). As a result of its relative recency, long COVID is not well-defined epidemiologically nor is the pathophysiology understood. In the years to come long COVID will impose new burdens on healthcare systems, making it urgent to develop new tools to manage the multiple dimensions of the disease. Underscoring the importance of this challenge, the Spanish Society of Cardiopulmonary Rehabilitation foresees an eventual collapse of its care systems due to the management of the cardiovascular sequelae of COVID-19 (5). A recent study of 5 million patients revealed that COVID-19 survivors experienced a significant increase (up to 2,000%) in the risk of suffering from cardiovascular (infarction, arrhythmias), pulmonary (hypoxemia, dyspnea), metabolic (diabetes, dyslipidemia) and neurological (cognitive impairment, sleep disorders, cerebral infarction) conditions from 1 to 6 months post-infection (1), with the highest risk observed in patients who were critical, followed by hospitalized and asymptomatic patients (3). Subsequent studies have shown a relationship between cardiovascular sequelae of COVID-19 and development of dysautonomia (6), often a product of chronic systemic inflammation that increases sympathetic nerve activity (6–8). This dysautonomia is a component of “post-COVID Guillan-Barré syndrome” (PCGBS) which is the most recurrent type of neurological post-COVID disorder (observed in 15% of patients) (8–11) and has been linked to the neuro-psychological sequelae of long COVID, such as anxiety, depression, and cognitive impairment (9, 10). Despite the fact that long COVID has not yet been fully characterized, dysautonomia is thought to play an important role in the pathophysiology of the syndrome (11, 12), especially with respect to the cardiovascular and neurological aspects. Accordingly, interventions intended to restore normal sympathovagal function could improve the cardiovascular and neurological complications of long-COVID (13, 14). With this in mind, we propose that cardiovascular rehabilitation programs (CRPs) are a feasible tool already established in clinical practice which may be applied to treatment of cardiovascular and neurological sequelae of long COVID.

Cardiovascular rehabilitation programs (CRPs) are interdisciplinary and multidimensional interventions that are defined by: (i) the recurrent execution of simple and well-tolerated exercises that stimulate parasympathetic activity and reduce sympathetic activity, (ii) a family education-based program of exercise, self-care and healthy habits promotion, (iii) an accompaniment program for patients and their caregivers (13). The benefits of CRPs for improving cardiorespiratory fitness has been consistently shown in large cohort studies, and they are first-line therapies for rehabilitation after myocardial infarction and stroke, as well as in the management of elder people with elevated cardiovascular risk (13–19). Given the effectiveness and feasibility of CRPs in clinical contexts, they have great promise as an approach for managing cardiovascular sequelae of long-COVID.

Therefore, we aimed to summarize post-COVID-19 cardiovascular consequences and to encourage health care professionals and private/public health organizations to evaluate/implement non-invasive techniques for the management of COVID-19 cardiovascular sequelae. For that, we selected clinical studies from 2020 to 2022 using the keywords “long-COVID,” “cardiovascular,” “autonomic/dysautonomia,” and other publications from 2009 to 2022 regarding to “cardiorespiratory fitness,” “cardiovascular rehabilitation,” “dysautonomia.” Finally, these studies were filtered according to their pertinence for long-COVID cardiovascular sequelae epidemiology and non-invasive strategies to improve cardiovascular and autonomic outcomes of long-COVID syndrome, as well as their potential feasibility in clinical contexts. This resulted in the selection of 54 publications, including epidemiological studies, clinical trials, scientific papers, and reviews.

## 2. Long-COVID syndrome pathophysiology

**FIGURE 1:**
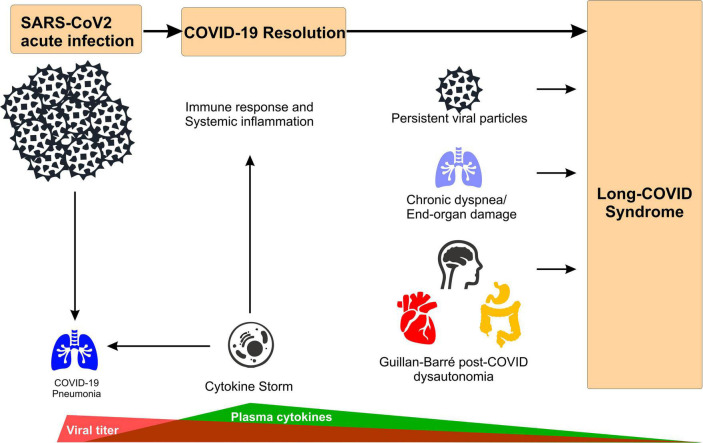
Main mechanism of long-COVID syndrome. Acute SARS-CoV-2 infections leads to broad inflammatory response (i.e., cytokine storm) to combat viral infection that causes COVID-19 pneumonia. After infection resolution, potential mechanisms underlying long-COVID syndrome include: (i) remanent viral particles in several tissues/organs, (ii) chronic dyspnea associated with lung function impairment (i.e., hypoperfusion, focal ischemia), and (iii) Guillan-Barré-like dysautonomia post-COVID, characterized by depression/anxiety behavior, excessive daytime fatigue, cardiac arrhythmogenesis, orthostatic hypotension and digestive system complications.

COVID-19 is caused by the severe acute respiratory syndrome coronavirus 2 (SARS-CoV-2). Most of the patients affected by the acute form of the disease (i.e., acute COVID-19) develop mild symptoms such as anosmia, fever, headache, cough, fatigue, and muscle aches (Figure 1 and Table 1; 20). However, a more susceptible population can also develop a severe pneumonia, with the most severe cases progressing to respiratory failure and death (21, 22). Even though SARS-CoV-2 is considered a respiratory virus, COVID-19 disease is a complex inflammatory syndrome that causes diffuse peripheral organ damage that has been shown to adversely affect myocardial, renal, gastrointestinal, and neurological function. In approximately 30% of the cases, neurological dysfunction may include demyelination, encephalitis, encephalopathies, hallucinations, and general behavioral alterations (Figure 1 and Table 1; 23–25). Strikingly, current evidence indicates that up to 50% of COVID-19 patients could develop a post-acute syndrome after the original SARS-CoV-2 infection (26, 27), while one study indicates that 87% of patients continue expressing at least one sign of the disease over 2 months after the first infection (28). Other common symptoms of long COVID include memory loss, alteration of taste and smell, muscle pain and sleep disorders, along with signs specifically associated with autonomic nervous system-dysfunction and related cardiovascular abnormalities (i.e., tachycardia, palpitations, chest pain, thromboembolism, myocardial fibrosis, inflammatory heart disease, and cerebrovascular disorders) (Figure 1 and Table 1; 2, 25, 27, 29).

**TABLE 1:** Main characteristics of acute-COVID and long-COVID syndrome*.

	Acute COVID-19	Long-COVID syndrome
Time course window	1 to 4 weeks	4 weeks to > 6 months
Viral detection by PCR	Positive	Negative
Common respiratory symptoms	Dyspnea Cough Sore throat	Dyspnea Cough Oxygen requirement
Major non-respiratory symptoms	Fever Fatigue, muscle/Body aches Headache Loss of taste and/or smell	Fatigue, muscle weakness, joint pain Sleep disturbances Cognitive impairment (“brain fog”) Tachycardia, palpitations, chest pain, inflammatory heart disease Chronic kidney failure Inflammatory bowel disease
Causes (major leading hypothesis)	SARS-CoV2 infection	Lung hypoperfusion (blood cloths) Persistent viral proteins/RNA Persistent inflammation

*Based on Nalbandian et al. (27) Nat Med 27:601–615; Couzin-Frankel (36) science, 376:1261–5, and World Health Organization and Mayo Clinic data base on COVID-19 disease.

Patients suffering long COVID do not necessarily test positive for SARS-CoV-2 *via* PCR detection, even in the early first infection phase, and it seems that the risk for developing long-COVID does not correlate with the severity of the acute phase of the virus (30, 31). Although the etiology(ies) of the long-COVID syndrome is still undetermined, there are several reports indicating the presence of chronic cardiorespiratory impairment (i.e., a chronic decrease in lung blood flow) and a hyperinflammatory state (32–34).

Three primary non-exclusive hypotheses have emerged as the possible causes of long-COVID:

1. The first revolves around end-organ vascular endothelial dysfunction and related hypercoagulability leading to microvascular thromboses and local ischemia. Evidence from lung blood flow measurements suggest a chronic impairment in lung vessels due to the presence of small blood clots in pulmonary capillaries and arterioles, leading to hypoperfusion, V/Q mismatch, hypoxemia, and the consequent chronic dyspnea observed in the long-COVID syndrome (28, 35).
2. The second hypothesis theorizes that the persistence of SARS-CoV-2 viral particles either embedded in organ tissue or spread systemically *via* extracellular vesicles stimulates a persistent or intermittent inflammatory response ultimately promoting thromboses and local organ dysfunction. The presence of SARS-CoV-2 viral particles in the lung, brain and heart in post-mortem tissue analysis has been observed (35, 36). Unfortunately, most of this data comes from studies that did not differentiate between acute and long-COVID diseases. However, a recent report indicates the presence of viral particles more than 6 months after the first mild acute-COVID-19 manifestation in long-COVID patients (36, 37). In the study, 70% of the individuals suffering inflammatory bowel disease, presented RNA and proteins of the virus in the gut tissue. Although the causal relationship between these “lingering” viral particles and the development of long-COVID symptoms remains to be elucidated, this evidence invites speculation on whether these viral molecules are responsible for the hyperinflammatory response present in the chronic form of the disease (32, 36).
3. Finally, several studies report that the uncontrolled inflammatory response broadly described in COVID-19 patients (i.e., systemic cytokine storm) (32, 34) might be linked to a hyperactive immune system response, which can be altered even up to 8 months after the initial infection (Table 1). Relatedly, there is some thought that acute COVID-19 might spur long-term autoimmune dysfunction such as PCGBS leading to neural degeneration, autonomic dysregulation, and related organ system dysfunction.

One, or more likely, a combination of these hypotheses could explain the causes of this chronic form of COVID-19. Further research is still necessary to determine the origin and the precise mechanisms underlying long-COVID syndrome.

## 3. Cardiovascular and autonomic consequences of long-COVID

It has been proposed that long-COVID emerges as a consequence of remnant viral particles after acute COVID infection that drives a sustained systemic inflammatory response, which in turn drives cardiovascular, respiratory, neurological, and/or metabolic sequelae (11). Importantly, not every patient experiences the same long-COVID syndrome, depending on their particular inflammatory response after SARS-CoV-2 infection.

One study revealed higher risk of developing cardiovascular diseases up to 12 months post COVID-19 infection compared to contemporary controls (a cohort of more than 5 million people with no evidence of SARS-CoV-2 infection during the study period) and historical controls (additional cohort of ∼6 million people during 2017) (2). These cardiovascular diseases included cerebrovascular diseases (stroke and transient ischemic attacks), dysrhythmias (atrial fibrillation, sinus tachycardia/bradycardia, ventricular arrhythmias, and atrial flutter), cardiac inflammatory disease (pericarditis and myocarditis), ischemic heart disease (acute coronary disease, ischemic cardiomyopathy, and myocardial infarction), heart failure, thromboembolic disorders, and non-ischemic cardiomyopathy; independent of pre-existing cardiovascular morbidities (2). The results showed that the adjusted incident rate ratios of cardiovascular outcomes in the post-COVID-19 exposure period were significantly higher than those in the pre-exposure period and exhibited a graded increase by severity of the acute phase of the disease, and importantly, vaccination significantly reduced the risk of developing myocarditis and pericarditis, supporting the notion that cardiovascular consequences of SARS-CoV-2 infection are dependent on viral infection *per se* rather than pre-existing comorbidities (2).

In the context of cardiovascular and autonomic consequences of long-COVID, it has been reported that around 13% of acute COVID and long-COVID infected patients show a specific type of dysautonomia termed “post-COVID Guillan-Barré syndrome” (PCGBS) (3), described as a microinflammation exclusively occurring in autonomic nerve fibers (vs. autonomic and motor fibers in “traditional” Guillan-Barré syndrome). This localized inflammation drives nerve constriction and augments basal autonomic fiber activity promoting chronic activation of the sympathetic nervous system. This in turn leads to arrhythmogenesis, orthostatic hypotension, altered peristalsis and/or cognitive decline (11). Also, PCGBS has been reported among the fatal complications of long-COVID (30). The reasons why PCGBS is purely autonomic are not known, but it is accepted that dysautonomia (especially chronic sympathetic activation) could be a central focus in the management of long-COVID patients, given the role of the autonomic nervous system in cardiac, respiratory, and metabolic function (3). Therefore, the application of strategies aiming to restore normal autonomic nervous system activity, such as CRP, could have a positive impact on cardiovascular and sympathetic consequences of long-COVID.

## 4. Diagnostic approaches to long-COVID dysautonomia

There are several direct and indirect clinical tools for diagnosing dysautonomia, including measurements of plasma catecholamines, heart rate variability analysis (HRV), spontaneous baroreflex sensitivity analysis, skin sympathetic nerve activity, skeletal muscle microneurography, and the COMPASS-31 survey, among others (2, 26). Measurement of plasma catecholamines would be considered a gold standard direct measurement of autonomic activation however this requires a blood draw and additional laboratory testing. The COMPASS-31 survey can easily be applied to patients, but it has several notable limitations, most importantly that it does not generate a quantitative score and that it is dependent on patient recall and honesty. For these reasons its value and application in clinical contexts has been questioned. HRV is a more suitable tool for diagnosing post-COVID dysautonomia compared to COMPASS-31 given that it generates a quantitative score, it is non-invasive, its application is independent of consciousness or cognitive function, it does not rely on patient recall or honesty, and it has been robustly validated in clinical practice as indicator of autonomic function (38–40). In fact, a pilot study has validated HRV analysis as a predictor for the inflammatory and autonomic state of post-COVID patients by using short ECG recordings and AI-processing, making it a potentially powerful tool for diagnosing long-COVID dysautonomia and predicting related cardiovascular dysfunction (41).

## 5. Cardiac rehabilitation programs as a therapeutic adjunct in treatment of long-COVID

Recently CRP has emerged as a potential tool for managing cardiorespiratory and autonomic dysfunction associated with long-COVID. In a pilot study undertaken in Japan (*n* = 50, 65–74 years of age), a CRP program including easy cardiovascular rehabilitation exercises, education, and individual psychosocial support program, resulted in 90% adherence, a significant reduction in anxiety, improved patient autonomy, and a positive impact on patient quality of life (42). A recent case study examined the efficacy of a personalized CRP in a patient with confirmed PCGBS, and found dramatic improvements in dyspnea, fatigue, muscular strength, autonomy, and functional state (43). These early studies suggest that the application of personalized CRP in patients with long-COVID is a feasible and potentially effective approach to managing autonomic and cardiovascular sequelae of long-COVID.

## 6. Clinical management of long-COVID-associated cardiovascular dysfunction

As previously discussed, several cardiovascular complications have been described in patients with COVID; however, there is still much to discover about these complications in post-COVID patients, and even more in those patients who have long-COVID. The WHO and the Long-COVID Forum Group have declared the importance of studying and clinically characterizing long-COVID patients to be able to create care and management strategies for these patients in the future (2). Healthcare organizations have stated that research priorities should aim to identify characteristics of long-COVID however given the diffuse and varied presentation of this condition this will undoubtedly be a challenging task (44). What is known at the present time is that there is a wide range of cardiovascular manifestations associated with long-COVID (i.e., those directly related to COVID-19 infection such as pericarditis and myocarditis; and the other ones plausibly related to systemic inflammation and PCGBS, including dysautonomia, arrhythmias, fatigue), and therefore a wide range of potential treatments. In order to tailor potential treatments continuous observation of cardiac biomarkers could be used to fine-tune treatment strategies to the specific manifestation of long-COVID in any given cohort (44). In support of this notion, an expert panel recently convened by the American College of Cardiology recommended that all patients who have had COVID-19 should be tested for abnormal cardiac function especially those with known immunosuppression and older adults at risk for suffering adverse cardiovascular events associated with long-COVID (45). Whether alterations in cardiac function cause or result from impaired cardiac autonomic regulation is still not known. However, the NICE guidelines recommend the use of β-blockers for angina, coronary syndromes, and cardiac arrhythmias, suggesting that controlling for cardiac sympathoexcitation in long-COVID may offer therapeutic potential in this population (44). An important factor to consider when designing an intervention in long-COVID patients is age. There is evidence that, depending on the age of those involved, COVID-19 infection can result in significant cardiovascular events such as subclinical myocarditis (46). Due to the large number of factors that could mask symptoms in long-COVID patients, it is imperative to develop effective screening, and specialized care and treatment programs. There are clinical studies that have tested different treatment regimens for long-COVID, such as medications, dietary supplements, and even the use of hyperbaric oxygen, but the appropriate design(s) for clinical management still is undetermined (47). There is ample evidence that exercise training improves cardiovascular and autonomic function in clinical populations with underlying cardiac dysfunction (48–50). With this in mind, we are enthusiastic that exercise may represent a complementary therapeutic strategy that may be beneficial to long-COVID patients with dysautonomia and cardiovascular dysfunction (47). These benefits may accrue through a variety of pathways including improvements in vascular endothelial function, autonomic function, and direct effects on myocardial function (51). Focused future studies are needed to provide compelling and comprehensive evidence that support incorporation of exercise programs in the treatment of autonomic and cardiovascular dysfunction associated with long-COVID.

## 7. Long-COVID syndrome and children

Most investigations of acute and long-COVID infection have been focused on older adults due to their high vulnerability to adverse events. Few studies have analyzed the pediatric COVID-19 population (52). It is recognized that available data on the pediatric population should be interpreted with caution since it’s still incomplete and/or missing adjusted values according to several confounding factors (52). Long-COVID-like syndrome has been reported in children and adolescents from 4 to 15 years old (53). Children with SARS-CoV2 history present identical symptoms to those present in Kawasaki disease, cytokine storm, or toxic shock syndrome. Initially, this new set of symptoms were named “Kawashocky” or “pediatric COVID-19 associated inflammatory disorder” (53). Later, The Royal College of Pediatrics and Child Health defined it as “pediatric inflammatory multisystem syndrome temporally associated with SARS-CoV-2” (MIS-C) (54). Interestingly, the underlying mechanisms of MIS-C and adult long-COVID are similar, involving systemic inflammation, cytokine storm, and oxidative stress. MIS-C symptoms have been observed to start from 2 to 6 weeks after acute SARS-CoV2 infection and include cardiac dysfunction and dyspnea (52–54); however, no data from autonomic sequelae in infants/adolescents are currently available. Furthermore, no comprehensive follow-up studies have been done in the pediatric population after COVID infection. This precludes any definite conclusion about any mechanisms that may be involved in cardiovascular/autonomic sequelae of long-COVID in this population. Accordingly, there is an urgent need for studies in children/adolescents to fully characterize long-term sequelae of COVID-infection in order to provide clinical management strategies specially designed for this population that may help to improve long-term outcomes.

## 8. Conclusion

In summary, long-lasting cardiovascular sequelae of COVID-19 infection are partially mediated by alterations in the autonomic nervous system. Accordingly, the application of new cardiovascular rehabilitation programs to the clinical management of long-COVID patients should provide healthcare personnel with the ability to manage the consequences of long-COVID and may help to reduce future disease burden.

## Data availability statement

The original contributions presented in this study are included in the article/supplementary material, further inquiries can be directed to the corresponding author.

## Author contributions

FA wrote first draft. HD, FO, NM, RQ, NI, and RDR contributed to manuscript formulation and revision. All authors have read and approved the final manuscript.
